# Does Plasminogen Activator Inhibitor-1 Drive Lymphangiogenesis?

**DOI:** 10.1371/journal.pone.0009653

**Published:** 2010-03-11

**Authors:** Françoise Bruyère, Laurence Melen-Lamalle, Silvia Blacher, Benoît Detry, Anne Masset, Julie Lecomte, Vincent Lambert, Catherine Maillard, Gunilla Høyer-Hansen, Leif R. Lund, Jean-Michel Foidart, Agnès Noël

**Affiliations:** 1 Laboratory of Tumor and Development Biology, Groupe Interdisciplinaire de Génoprotéomique Appliqué-Cancer (GIGA-Cancer), University of Liège, Liège, Belgium; 2 Department of Ophthalmology, Centre Hospitalier Universitaire (CHU), Liège, Belgium; 3 The Finsen Laboratory, Rigshospitalet, Copenhagen, Denmark; 4 Department of Gynecology, Centre Hospitalier Universitaire (CHU), Liège, Belgium; Karolinska Institutet, Sweden

## Abstract

The purpose of this study is to explore the function of plasminogen activator inhibitor-1 (PAI-1) during pathological lymphangiogenesis. PAI-1, the main physiological inhibitor of plasminogen activators is involved in pathological angiogenesis at least by controlling extracellular proteolysis and by regulating endothelial cell survival and migration. Protease system's role in lymphangiogenesis is unknown yet. Thus, based on its important pro-angiogenic effect, we hypothesized that PAI-1 may regulate lymphangiogenesis associated at least with metastatic dissemination of cancer cells. To address this issue, we studied the impact of PAI-1 deficiency in various murine models of tumoral lymphangiogenesis. Wild-type PAI-1 proficient mice were used as controls. We provide for the first time evidence that PAI-1 is dispensable for tumoral lymphangiogenesis associated with breast cancers either induced by mammary carcinoma cell injection or spontaneously appearing in transgenic mice expressing the polyomavirus middle T antigen (PymT) under the control of a mouse mammary tumor virus long-terminal repeat promoter (MMTV-LTR). We also investigated inflammation-related lymphatic vessel recruitment by using two inflammatory models. PAI-1 deficiency did neither affect the development of lymphangioma nor burn-induced corneal lymphangiogenesis. These novel data suggest that vascular remodelling associated with lymphangiogenesis and angiogenesis involve different molecular determinants. PAI-1 does not appear as a potential therapeutic target to counteract pathological lymphangiogenesis.

## Introduction

The lymphatic network is composed of blind-ended lymphatic vessels that regulate tissue homeostasis, the afferent immune response and fat transport. After collecting extravasated protein-rich fluid and lymphocytes from the extracellular space or triglycerides from the gut, lymphatic capillaries transport them back to the blood circulation through larger vessels and lymph nodes [1]. Tumoral cell metastasis may occur by invading either the blood circulation or the lymphatic vascular system. The increased permeability and the absence of basement membrane of lymphatic vessels compared to blood vessels facilitate the intravasation of tumor cells into the lymphatic system. Therefore, lymphatic vessels offer an easy way for cancer cells to disseminate and form metastasis into the lymph nodes before reaching the blood circulation. This is supported by the clinical investigation of the sentinel lymph node while searching for cancer dissemination in patients [2]. Moreover, the lymphatic system is involved in chronic inflammatory diseases and in transplant rejection (renal and corneal graft rejection) [3]. Therefore, a better understanding of the molecular and cellular basis of lymphatic abnormalities associated with cancers and inflammation is essential for the development of novel therapeutic strategies.

Components of the plasminogen activation system are involved in several physiological and pathological processes associated with important tissue remodelling [4]. This proteolytic system is composed of serine proteases including tissue-type plasminogen activator (tPA), urokinase-type plasminogen activator (uPA), uPA receptor (uPAR) and the plasminogen activator inhibitors (PAI), the main one being PAI-1. The contribution of these proteins in cancer progression relies on their capacity to control various biological processes such as (i) cell proliferation [5]; (ii) cell invasion through plasmin-mediated extracellular matrix degradation [6]; (iii) cell adhesion and migration through the interaction of uPA/uPAR/PAI-1 complex with vitronectin and integrins [7]; and (iv) cell apoptosis through the control of pro-apoptotic factor release [8]. PAI-1 is now recognized as an essential factor of the host microenvironment that promotes tumor growth, vessel recruitment [9]–[14] and dissemination of tumoral cells to distant organs [15], [16]. The effect of PAI-1 depends on its concentration, being pro-angiogenic at physiological concentration and anti-angiogenic at pharmacological concentrations [14], [17]–[19] These experimental observations confirm the positive correlation existing between PAI-1 blood levels, the rate of metastasis and the poor prognostic of patient with different types of cancers (for review: [5]). The essential role of PAI-1 in angiogenesis was also demonstrated in a laser-induced model of choroidal angiogenesis that mimics the age-related macular degeneration extending its function to ocular diseases [11], [14].

Based on its pivotal role during tumoral angiogenesis, a contribution of PAI-1 in lymphangiogenesis is anticipated, but not documented. In the present study, to address this issue, we applied different models of tumoral and inflammation-induced lymphangiogenesis into PAI-1 deficient mice having different genetic backgrounds.

## Methods

### Transgenic Modified Mice

Homozygous PAI-1 deficient mice (PAI-1^−/−^) and their corresponding wild type mice (PAI-1 WT) were those previously used for angiogenesis studies with a mixed genetic background of 87% C57BL/6 and 13% 129SV/SL strain [8], [17], [20]. Their corresponding immunodeficient mice were generated in a RAG-1^−/−^ background and genotyped by PAI-1 and PGKPA gene annealing as previously described [10], [20]. Heterozygous FVB/N-PyMT male mice obtained from Finsen Laboratory (Copenhagen, Denmark) were mated with homozygous PAI-1^−/−^ and PAI-1 WT female mice, which were backcrossed for eight generations into the FVB/N strain [21]. Mouse experimentation was done in accordance to the guidelines of the University of Liège regarding the care and use of laboratory animals.

### Cell Lines

The human MCF-7 breast carcinoma cell line transfected either with human VEGF-C mature form cDNA (MCF-7/VEGF-C) or with control cDNA (MCF-7/EB57II) were obtained from Kari Alitalo (University of Helsinki, Finland) [22]. Cells were routinely cultured in RPMI-1640 medium (Invitrogen, Carlsbad, CA) supplemented with GlutaMAX, 25 mM Hepes, penicillin (100 U/ml), streptomycin (100 mg/ml), hygromycin (150 mg/ml) and 10% fetal bovine serum (Gibco, Grand Island, NY). Amplification of VEGF-C cDNAs was carried out by polymerase chain reaction using the reverse primers: TTG-TTC-GCT-GCC-TGA-CAC-TG and the forward primers: CTC-TCT-CTC-AAG-GCC-CCA-AA. The PCR condition was 94°C for 0.15 min, 55°C for 0.2 min, and 72°C for 0.2 min, for 30 cycles. Human VEGF-C ELISA (R&D Systems, Minneapolis, MN) was performed on medium conditioned by cells according manufacture's instructions.

### Mammary Orthotopic Transplantation

Subconfluent MCF7 cultures were harvested by trypsinization, washed twice and resuspended in serum-free medium. Cells (2.5×10^6^/50 µl) were bilaterally inoculated into the fat pads of the fifth mammary glands (mfp) of 8- to 12-week-PAI-1^−/−^ and, -WT RAG-1^−/−^ female mice. Mice were supplemented with a subcutaneous 60-days slow-release pellet containing 0.72 mg of 17ß-estradiol (Innovative Research of America, Toledo, OH). Tumor growth was measured with a calliper and the volume was calculated according to the formula V = (π/6)(d_1_Xd_2_)^3/2^ [23]. After 16 weeks, mice were killed and tumors, lungs and lymph nodes were collected. All the samples were formalin fixed and paraffin embedded. The tumor incidence is defined as the percentage of animals bearing a palpable tumor. Two separate sets of experiments with similar results were performed with each time at least 8 to 11 mice per experimental group; presented data are combined results (34–38 injected mammary fat pads (mfp) per condition).

### Tumor Processing in PyMT Mice

Female mice heterozygous for the PyMT transgene and homozygous for the wild type or mutated PAI-1 gene were used in these studies. After 8, 11 or 14 weeks, mice were killed and tumors, lungs and lymph nodes were collected. Tumors were weighted before formalin fixation and paraffin embedding. To avoid any variance from the fat pad localisation (peritoneal-localised tumors being bigger than those developed in the thoracic cavity), all the individual tumor weights were summed to give the total tumor weight of each mice. The number of mice sacrified per genotype was: 19 for PyMT PAI-1 WT mice and 23 for PyMT PAI-1^−/−^ mice sacrified at 14 weeks; 17 for PyMT PAI-1 WT mice and 9 for PyMT PAI-1^−/−^ mice sacrified at 11 weeks and 12 for PyMT PAI-1 WT mice, and 7 for PyMT PAI-1^−/−^ sacrified at 8 weeks.

### Lymphangioma

Lymphatic endothelial hyperplasia (lymphangioma) formation was induced by two intraperitoneal injections of incomplete Freund's adjuvant [24]. For ethical purposes, buprenorphine hydrochloride injections (0.05 mg/kg, TEMGESIC®, Reckitt Benckiser, UK) were administered every 12 h during the first 5 days 1 h before and after adjuvant injections. After 4 weeks, mice (n = 6) were killed and lymphangioma masses appearing at the surface of diaphragm were photographed for quantification using the ImageJ 1.40 g software (NIH). The lymphangioma development was determined by reporting the lymphangioma surface on the total organ area. The lymphatic origin of tumors was assessed by positivity for LYVE-1 as described above. Sirius Red staining was obtained by incubating the deparaffinised and ionised slides with 0.1% Sirius Red (Polyscience, Warrington, PA)/picric acid (1.15 g/100 ml). Slides were then rapidly incubated in 90% alcohol and in xylene before being mounted with Eukitt medium.

### Immunohistochemistry on Slides and Metastasis Detection

All reagents and procedure are summarized in Table 1. In brief, after deparaffination and rehydration, immunostainings were performed on 6 µm slides by first unmasking antigens and subsequently blocking endogenous peroxidases by 3% H_2_O_2_/H_2_O for 20 min. Nonspecific binding was prevented by a specific treatment before the incubation with primary and secondary (if needed) antibodies followed by incubation with a streptavidin/HRP complex (DAKO, Glostrup, Denmark, 1/500) at room temperature (RT) for 30 min. Slides were stained with 3-3′diaminobenzidine hydrochloride (DAKO, Glostrup, Denmark) for 3 min to 10 min, counterstained with hematoxylin and mounted with Eukitt medium for microscope observation. Omission of the first antibody served as negative control.

**Table 1:** Antibodies and procedure used for immunochemistry to detect metastasis on lung and lymph node slides, lymphatic vessels and inflammatory cells on primary tumors/lymphangiomas.

	Steps of immunohistochemistry			
Antibody used	Unmasking	Blockage	Primary antibodies	Secondary antibodies
**Detection of MCF7 tumor metastasis in lymph nodes and lungs.**	0.05% H_2_O_2_/trypsin, 40 min at 37°C	PBS/bovine serum albumin 0% (Fraction V, Acros Organics1, NJ), 1 hour	Mouse anti-human pS2 protein (1/50, 1h30 at RT) (DAKO, Glostrup, Denmark)	Goat anti-mouse Envision/HRP (30 min at RT) (DAKO, Glostrup, Denmark)
**Detection of MCF7 tumor metastasis in lymph nodes.**	0.05% H_2_O_2_/pronase, 10 min at RT	normal goat serum, 30 min	Rabbit anti-cytokeratin WSS (Wide Spectrum Screening) (1/500, 1 h at 37°C) (DAKO, Glostrup, Denmark)	Goat anti-rabbit/biotine (1/400, 30 min at RT) (DAKO, Glostrup, Denmark)
**Detection of MCF7 tumor metastasis in lungs.**	Target Retrieval Solution (DAKO, Glostrup, Denmark), 11 min at 126°C	H_2_O_2_/Universal Blocking Reagent (1/10, BioGenex, San Ramon, USA), 10 min	Rat anti-Ki-67 (1/50, 1 h at RT), (DAKO, Glostrup, Denmark)	Rabbit anti-rat/biotine (1/300, 30 podoplaninmin at RT), (DAKO, Glostrup, Denmark)
**Detection of PyMT tumor metastasis in lymph nodes.**	Citrate buffer (pH 6), 11 min at 126°C	H_2_O_2_/Universal Blocking Reagent (1/10, BioGenex, San Ramon, USA), 10 min	Rabbit anti-cytokeratin 8 (1/100, 1 h at RT) (Abcam, Cambridge, PA)	Goat anti-rabbit/biotine (1/400, 30 min at RT) (DAKO, Glostrup, Denmark)
**Detection of lymphatic vessels**	Target Retrieval Solution (DAKO, Glostrup, Denmark), 11 min at 126°C	PBS/bovine serum albumin 10% (Fraction V, Acros Organics, NJ), 1 h	Rabbit anti-Lyve-1 (1/1000, 1h30 at RT) (Upstate, Lake Placid, NY)	Goat anti-rabbit/biotine (1/400, 30 min at RT) (DAKO, Glostrup, Denmark)
**Detection of lymphatic vessels**	Citrate buffer (pH 6), 11 min at 126°C	normal goat serum, 30 min	Syrian hamster anti-podoplanin (1/1000, 1 h at RT) (Reliatech, Braunschweig, Germany)	Goat anti-syrian hamster/biotine podoplanin(1/500, 30 min at RT) (Jackson ImmunoResearch, podoplaninBaltimore, US)
**Detection of inflammatory cells**	Citrate buffer (pH 6), 11 min at 126°C	H_2_O_2_/Universal Blocking Reagent (1/10, BioGenex, San Ramon, USA), 10 min	Mouse anti CD45/biotine podoplanin(1/500, 1 h at RT) (Pharmingen, San Diego, USA)	Amplification with biotinylated tyramide (PerkinElmer, MA, USA)

RT = Room temperature.

Metastases in the lymph nodes and in lungs of mice bearing MCF7 tumors or PyMT mice were detected by immunostaining (Table 1). Results are expressed as the percentage of animals presenting at least one metastatic nodule.

### Computerized Vessel Quantification in Tumors and Lymphangioma

ImageJ software (NIH) was used to first manually delineate the immunolabelled vessels. We binarized images and optimized the threshold to highlight the vessels. For mammary tumors, the following parameters were determined on the whole tumor section: the relative vascular area (lymphatic vessel area reported to the total area of the tumor (µm^2^/µm^2^)), the number of sections of lymphatic vessels (reported to the total area of the tumor (1/µm^2^)) and the mean vessel area (lymphatic vessel area divided by the number of vessels (µm^2^)). For lymphangiomas, due to the variation in lymphangioma width associated with different fibrotic reaction, vessel quantifications are expressed by total vessel area (µm^2^) (at least 3 images were analyzed per sample (n = 6)).

### Corneal Assay

Corneal lymphangiogenesis was induced by thermal cauterization of the central cornea by using an ophthalmic cautery (OPTEMP II V, Alcon Surgial, Fort Worth, USA). Seven days later, mice were sacrificed, eyes were removed and corneas were flat mounted (n = 5, one flat mount per mice). Whole corneas were stained after fixation in ethanol 70%. Cornea whole mounts were blocked in 1,5% BSA-3% Gloria milk for 1 h at RT and incubated overnight with rabbit anti-LYVE-1 (1/600, a kind gift from Dr. K. Alitalo, Finland). After four 10 min washes with PBS, corneas were incubated with the FITC-coupled swine anti-rabbit Ig (1/40; DAKO, Glostrup, Denmark) for 60 min at RT and whole-mounted on a microscope slide with Vectashield-DAPI mounting medium (Vector Laboratories, Burlingame, CA). The area covered by immunostained lymphatic vessels was determined and normalized to the total corneal area. We also measured the length densities defined as the total area occupied by lymphatic network and the cumulative length of vessels. For both parameters, results are expressed per corneal area unit (µm^2^). As the mechanism of vascular growth is sprouting, its extent can be detected by the increase of vessel network extremities, which corresponds to the number of smallest vessels (so-called first order vessels in the centripetal ordering method) [25]. To achieve this, we determined the end-point density defined as the vessel network extremity number per corneal area unit. The branching density (defined as the vessel branching number per corneal area unit) was also measured to evaluate the degree of the vessel tree complexity [26].

### Statistical Analysis

Statistical analyses were performed using GraphPad PRISM 4 software. Incidences were evaluated as contingency table by Fisher's exact test. Other variables were tested by a one-way ANOVA (Kruskal-Wallis test) followed by the Mann Whitney test in order to avoid multiplicity. These tests were also used to determine significance in the morphometric analyses. Results are expressed ± S.E.M. Differences were considered statistically significant for *P* values less than or equal to 0.05.

## Results

### PAI-1 Is Dispensable for Lymphangiogenesis in an Experimental Model of Breast Cancer

**Figure 1:**
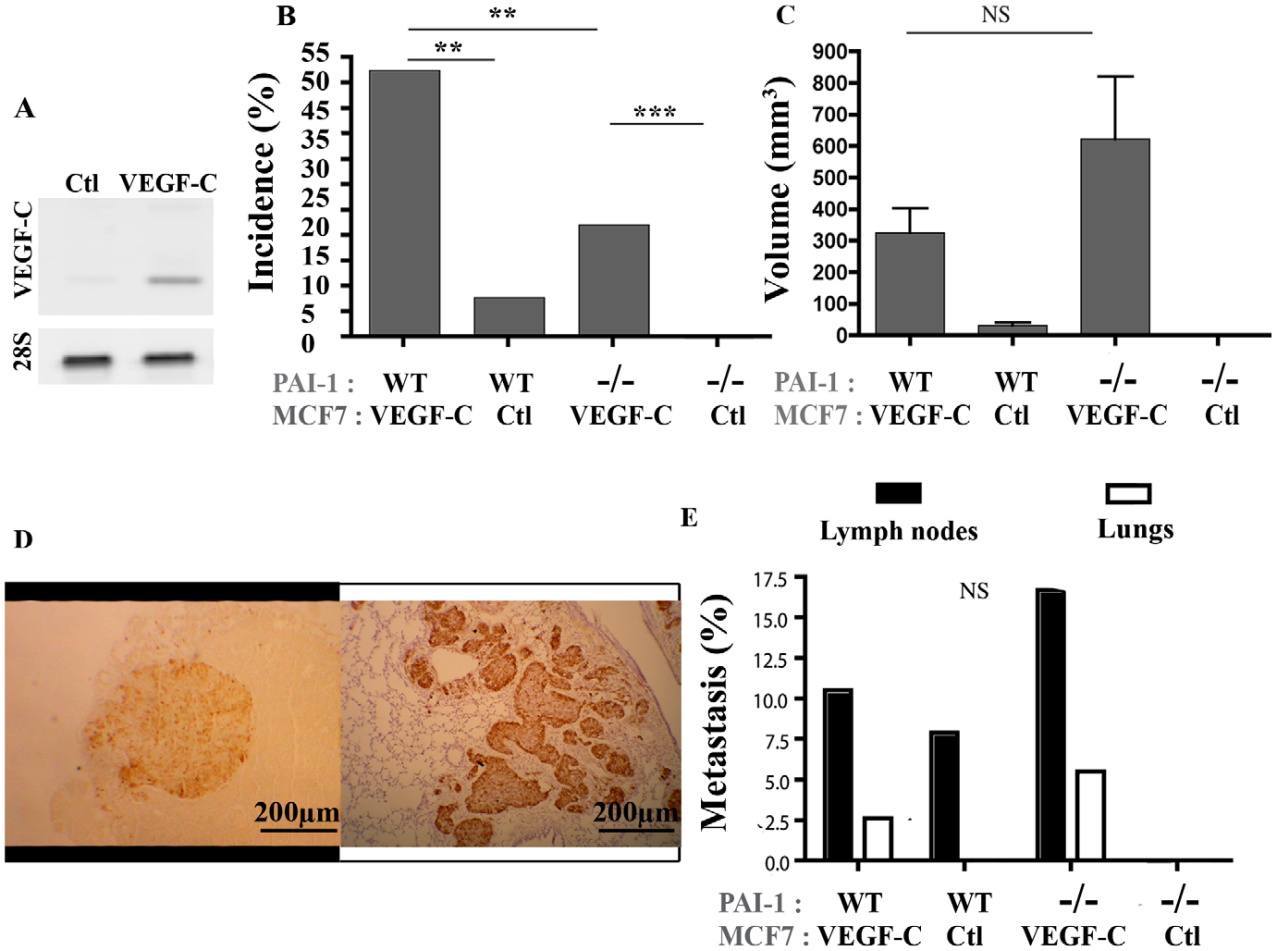
Tumor development after orthotopic injection of VEGF-C overexpressing MCF7 cells or control MCF7 cells implanted in the mammary fat pads (mfp) of PAI-1 WT or PAI-1^−/−^ mice. (A): RT-PCR analysis of VEGF-C and 28S mRNA expression by MCF7. (B): Tumor incidence (%) is defined as the percentage of palpable tumor per mfp. (C): Tumor volume was measured as described in Material and Methods. (D): Representative figure of a typical metastasis in lymph node (left) and lung (right). (E): Percentage of animal bearing at least a tumor nodule (metastasis% detected in lymph nodes (black boxes) and lungs (white boxes). Number of mfp per condition = 34–38. The mice PAI-1 status (WT or −/−) and the VEGF-C production (VEGF-C) or not (Ctl) by MCF7 cells are indicated below each graph. Data are ± S.E.M. Scale bars: 200 µm. ** P≤0.01, *** P≤0.001, NS = Non Significant.

To test the impact of PAI-1 on lymphatic vessel recruitment in tumors, VEGF-C-overexpressing MCF7 cells or control MCF7 cells were bilaterally injected into the fifth mammary fat pads (mfp) of immunodeficient RAG-1^−/−^ mice that are deficient in PAI-1 (PAI-1^−/−^) or not (PAI-1 WT). We checked the production of VEGF-C by the tumor cells in culture by RT-PCR (Fig. 1A) and in supernatants by ELISA (data not shown). Mice were supplemented with oestrogen by the subcutaneous implantation of oestradiol releasing pellets. Two separate sets of experiments with similar results were performed with at least 8 to 10 mice per experimental group (34–38 mfp per condition). At week 16, both PAI-1 WT and PAI-1^−/−^ mice implanted with VEGF-C expressing cells showed higher tumor incidence (*P* = 0.0051, *P*<0.0001, respectively; Fig. 1B) and, in PAI-1 WT mice, a tendency to a higher tumor volume (*P* = 0.1513; Fig. 1C) than mice inoculated with mock MCF7 cells. It is worth noting that the effect of VEGF-C on tumoral volume didn't reached the statistical significance due to the low number of tumors detected in mice injected with the non-VEGF-C producing cells (3 out of 38 mfp). Interestingly, PAI-1 deficiency reduced the incidence of tumors developed both in the presence of VEGF-C expression (52% of VEGF-C expressing tumors in PAI-1 WT mice *versus* 22% in PAI-1^−/−^ mice) (*P*<0.0089) and in its absence (8% of control tumors in PAI-1 WT mice *versus* 0% in PAI-1^−/−^ mice) (Fig. 1B). When considering the volume of tumors, differences did not reach statistical differences between PAI-1 WT and PAI-1^−/−^ mice (Fig. 1C).

The metastatic ability of cells was assessed by immunochemical staining with antibodies raised against pS2 protein and cytokeratin WSS on lymph node sections or against pS2 protein and Ki-67 on lung sections. Histological examination in the lung (*P* = 0.2879) or the lymph nodes (*P* = 0.1037) of mice bearing a VEGF-C expressing tumor revealed similar metastasis incidence for the two different groups (PAI-1 WT or PAI-1^−/−^ mice) (Fig. 1D, E).

**Figure 2:**
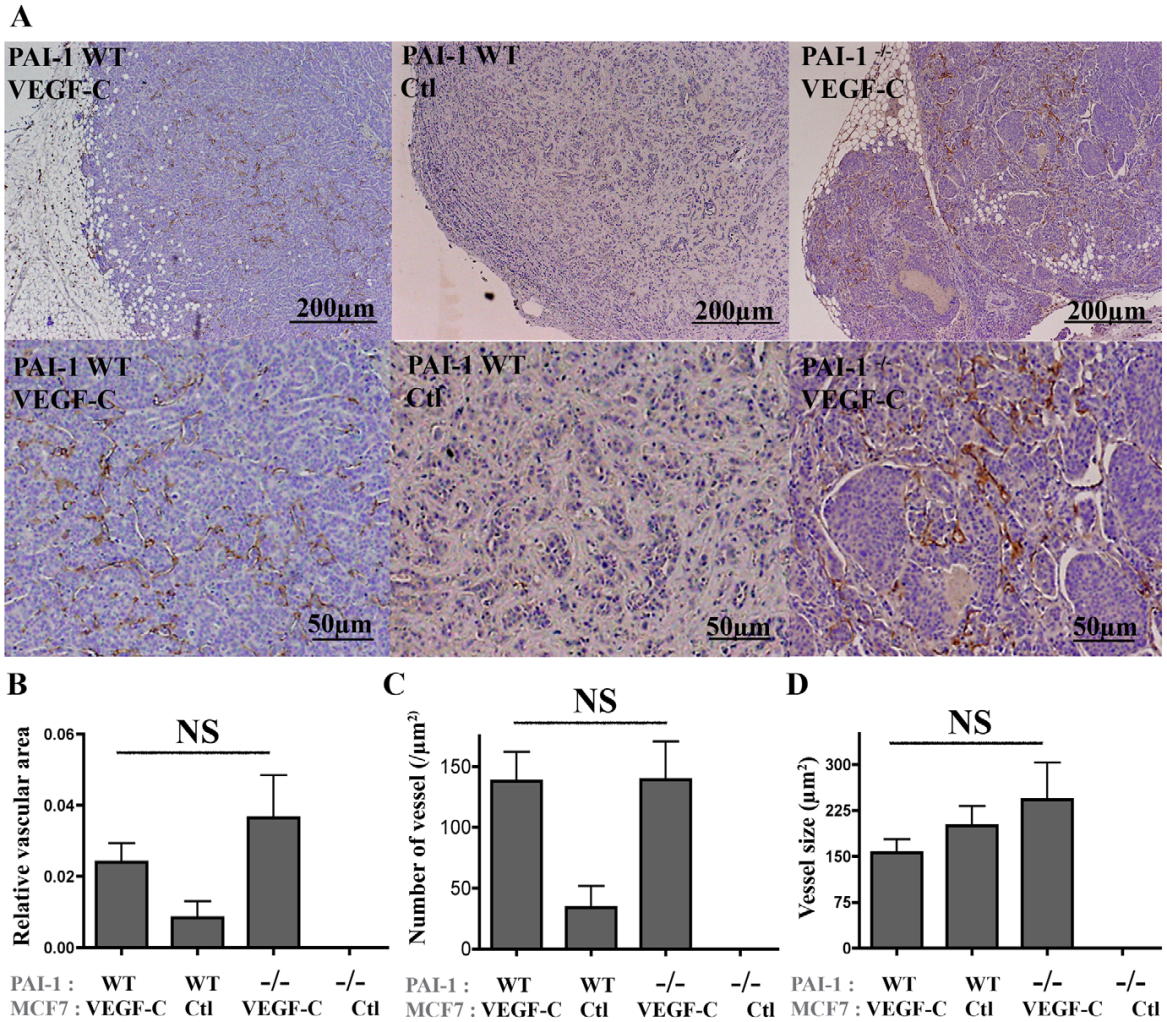
Analysis of intratumoral lymphatic vessels in tumors induced by orthotopic injection of VEGF-C overexpressing MCF7 or control MCF7 cells implanted in the mammary fat pads (mfp) of PAI-1 WT or PAI-1^−/−^ mice. (A): Representative images of Lyve-1 positive-vessels at two different magnifications in VEGF-C expressing tumors developed in WT mice (WT) or PAI-1 deficient mice (^−/−^), and in control tumors (Ctl). (B–D): Quantification of lymphatic vessels has been performed by computerized image analysis and led to the determination of three parameters: 1- relative vascular area (area occupied by intratumoral lymphatic vessels reported to the total tumor surface) (B); 2- number of lymphatic vessel sections per slide (number of vessels) (C) and; 3- mean lymphatic vessel size (D). The mice PAI-1 status (WT or −/−) and the VEGF-C production (VEGF-C) or not (Ctl) by MCF7 cells are indicated below each graph. Data are ± S.E.M. NS = Non Significant.

The lymphatic vasculature was next investigated on primary tumors (Fig. 2A). Through Lyve-1 immunolabeling on tumor sections, quantification performed by computerized image analysis led to the determination of 3 different parameters (Fig. 2B, C, D). In accordance to previous report [23], VEGF-C over-expressing tumors displayed a higher relative vasculature area than control tumors. However, the VEGF-C-induced lymphatic vasculature was similar in PAI-1 WT and PAI-1^−/−^ mice (*P* = 0.4048) (Fig. 2B). Because of the very low number of tumors developed in the absence of VEGF-C (3 out of 38 mfp in PAI-1 WT mice and 0 out of 34 mfp in PAI-1^−/−^ mice), no comparison could be performed between the two genotypes. This increased lymphatic vessel area observed in the presence of VEGF-C relied on an increased vessel number (Fig. 2C) while vessel size was unchanged (Fig. 2D).

### PAI-1 Is Dispensable for Lymphangiogenesis and Metastatic Dissemination in a Spontaneous Model of Breast Tumors

**Figure 3:**
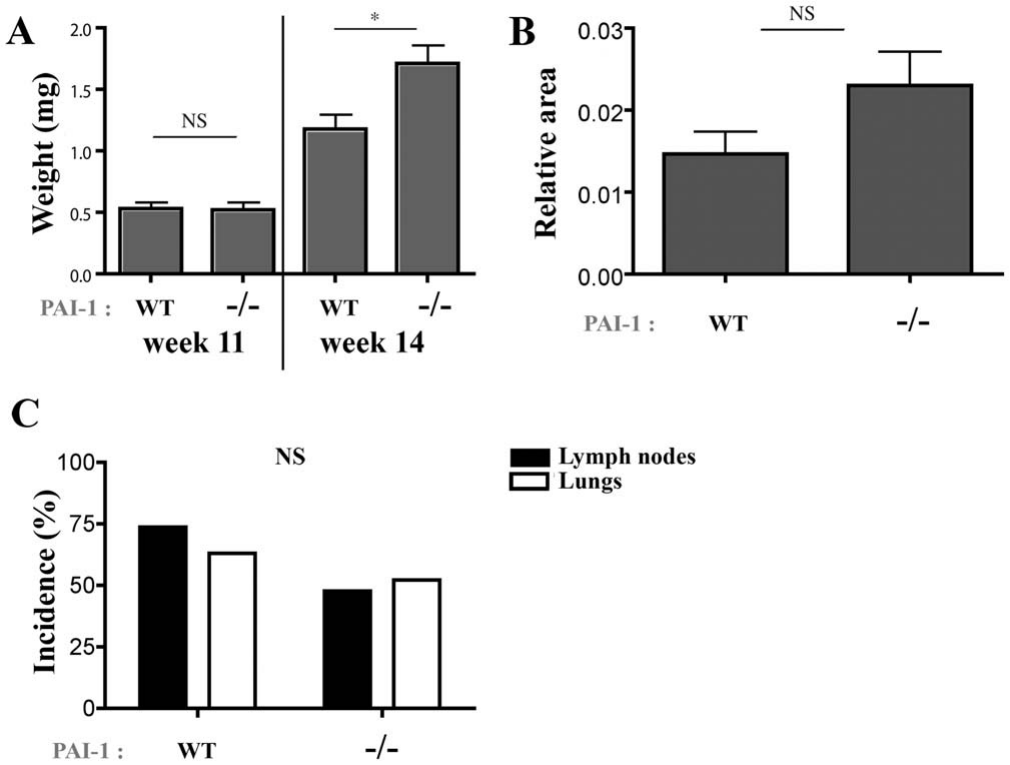
Primary tumor development and metastatic potential of PyMT mice crossed with PAI-1 WT or PAI-1^−/−^ mice. Presented results are from 11- and 14-week mice. (A): Tumor weight and (B): podoplanin positive relative surface in primary tumors observed in PAI-1 WT and PAI-1^−/−^ mice. (C): Percentage of metastasis in lymph nodes (black columns) and lungs (white columns). n = 16 PAI-1 WT-PyMT and 17 PAI-1^−/−^-PyMT mice at 14 weeks, 17 PAI-1 WT-PyMT and 9 PAI-1^−/−^-PyMT mice at 11 weeks. Data are ± S.E.M. *P≤0.05, NS = Non Significant.

In order to further analyse the role of PAI-1 in tumor growth and metastatic dissemination, we used transgenic PyMT mice that spontaneously develop mammary tumors. PyMT mice were crossed with PAI-1 WT or PAI-1^−/−^ mice in a FVB/N background. At week 8 or 11, difference was observed neither in tumor incidence (100%) nor in tumor weight (*P* = 0.8875; Fig. 3A). A very slight increase of tumor weight was even observed after 14 weeks in PAI-1 KO mice (*P* = 0.0461). Histochemical and immunochemical analyses detecting cytokeratin 8 and Ki67 did not reveal any difference in the metastatic dissemination into the lung or lymph nodes between PyMT PAI-1 WT and PyMT PAI-1^−/−^ mice sacrified after 14 weeks (fig. 3B). Indeed, metastatic nodules were detected in the lung of 63.1% (12/19) of PyMT PAI-1 WT mice and 52.2% of PymT PAI-1^−/−^ mice (12/23) (*P* = 0.5421). Similarly, lymph nodes of 73,68% (14/19) of PyMT PAI-1 WT mice and 47,8% (11/23) of PymT PAI-1^−/−^ mice (*P* = 0.1203) were infiltrated by tumoral cells.

In order to quantify the lymphatic vasculature inside primary PyMT tumors, a computer-assisted automatic quantification method was used [27]. No difference in terms of podoplanin labelling was observed between tumors generated in both genotypes after 14 weeks (*P* = 0.0945) (Fig. 3C).

### PAI-1 Deficiency Does Not Affect Inflammation-Related Lymphangiogenesis

**Figure 4:**
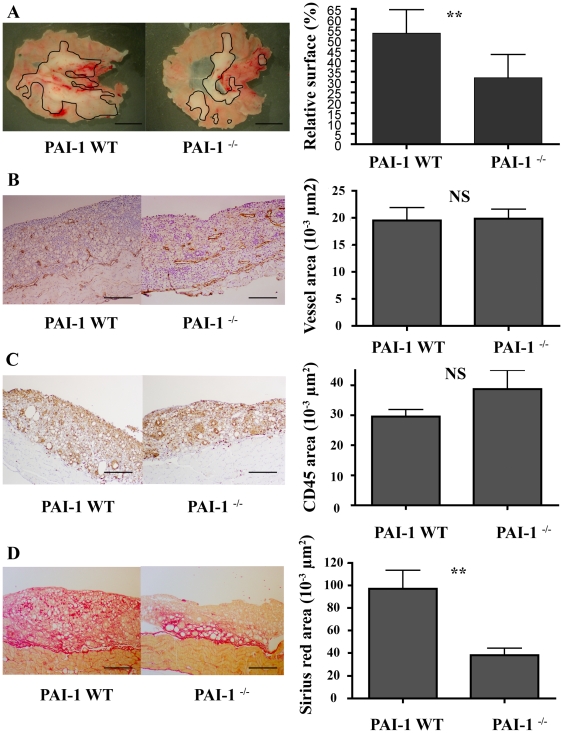
Development of lymphangioma in PAI-1 WT or PAI-1^−/−^ mice. A macroscopic decrease of lymphangioma is observed in PAI-1^−/−^ as compared to WT (A). Similar recruitment of lymphatic vessels (assessed by Lyve-1 positivity) (B), and of CD45 positive-inflammatory cells (C) was observed in both genotypes. Evaluation of fibrosis was performed by Sirius red staining (D). Representative images are shown on the left and quantifications performed by computerized image analysis are shown on the right Data are ± S.E.M (n = 6). Scale bars: A = 0.5 mm, B–D = 200 µm, ** P≤0.01.

In order to explore PAI-1 impact on the lymphatic vessel recruitment in inflammatory conditions, we applied a model of lymphatic endothelial cell hyperplasia called lymphangioma [24] to PAI-1 WT (n = 6) and PAI-1^−/−^ (n = 6) mice. After 4 weeks, mice were sacrified and the lymphangiomas that appeared as white masses underneath the diaphragm were measured. The surface of lymphangioma area was reported to the total area of the organ (percentage of invasion). Macroscopical analyses revealed that the lymphangioma surface was decreased with PAI-1 deficiency (*P*<0.01, Fig. 4A). Immunohistochemical analyses of these lymphangiomas were assessed with Lyve-1 and CD45 antibodies to label lymphatic vessels and inflammatory cells, respectively. Fibrosis was evaluated through Sirius red staining. Quantification was performed by a semi-automatic computer-assisted method. The microscopical analyses of lymphangioma sections showed that the area covered by lymphatic vessels was similar in PAI-1^−/−^ compared to corresponding wild type mice (*P* = 0.49, Fig. 4B). However, we pointed out differences in the lymphatic vessel structure between the experimental groups. The number of vessels per section was 54±5 µm^2^ in PAI-1 WT and 31±2 µm^2^ in PAI-1^−/−^ (*P* = 0.0002), and the vessel average surface was 383±43 µm^2^ in PAI-1 WT and was 663±57 µm^2^ in PAI-1^−/−^ (*P* = 0.0003). Thus, vessels in PAI-1^−/−^ mice were fewer but larger leading to an equal vessel area than counterparts. No difference was detected regarding the surface occupied by inflammatory cells (*P* = 0.44, Fig. 4C). In sharp contrast, decrease of red Sirius stained fibrosis was observed in PAI-1^−/−^ mice (*P*<0.01, Fig. 4D) and could explain the reduction of the lymphangioma area observed macroscopically in these mutant mice.

### PAI-1 Is Dispensable for Corneal Lymphangiogenesis

**Figure 5:**
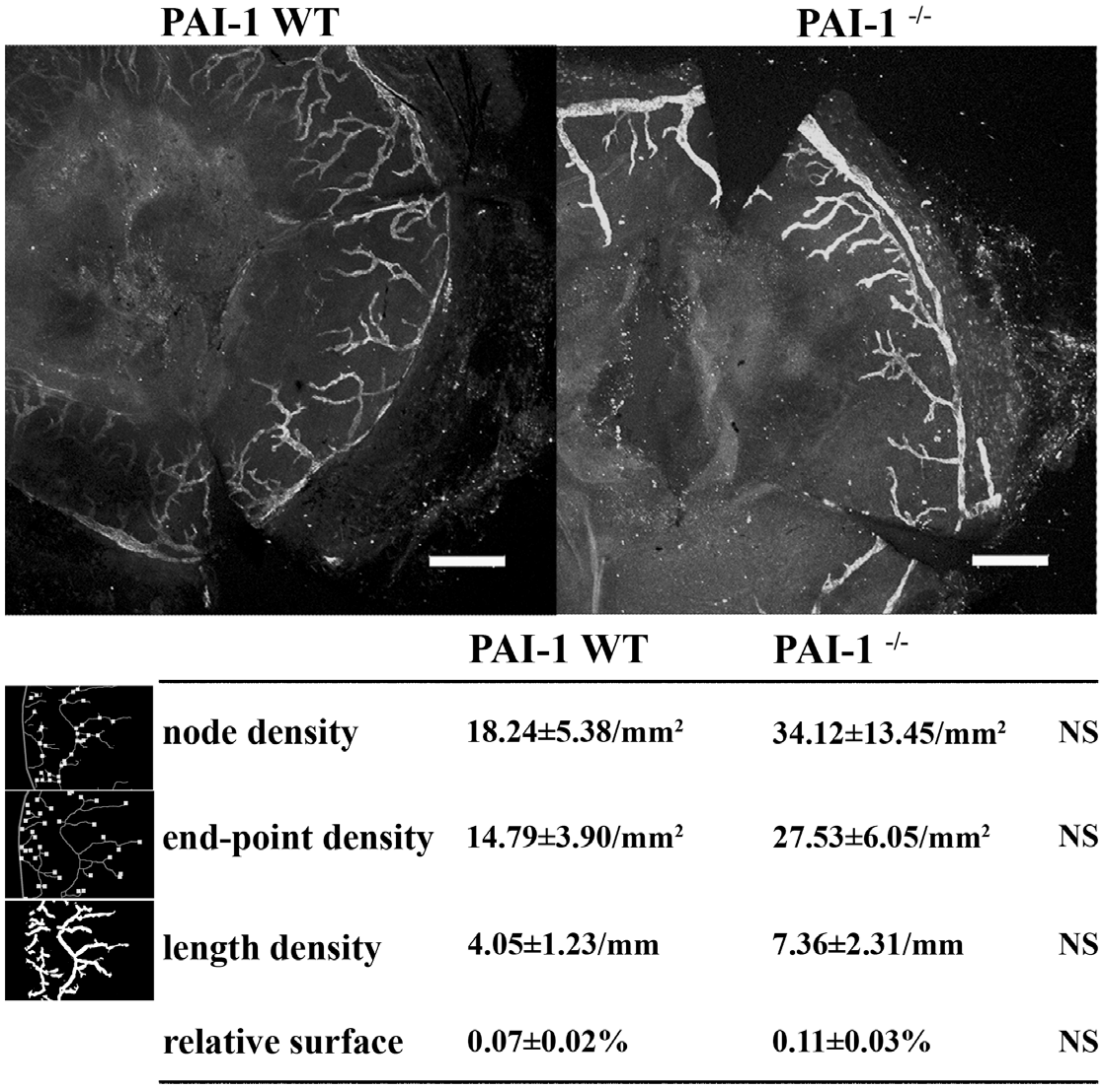
Photography and quantification of the Lyve-1 positive-network observed in the corneal assay in PAI-1 WT or PAI-1^−/−^ mice. The node, end-point and length densities are presented for both experimental groups with a schematic illustration of each of these measured parameters at the left. The area covered by the lymphatic vessels normalized to the total corneal area (relative surface, %) is also indicated. These parameters quantified by computerized image analysis are defined in Material and Methods. Data are ± S.E.M (n = 5). Scale bar = 400 µm, NS = Non Significant.

We then extended our study to an additional model of lymphangiogenesis related to inflammation. The corneal assay is an *in vivo* model which consists in a neovascularization induced by burning the central area of mouse cornea [28]. We previously confirmed the presence of PAI-1 mRNA in corneal lesions (data not shown). No modification of the lymphatic vessel recruitment was observed in the PAI-1^−/−^ mice (n = 5) compared to the wild type mice (n = 5) as assessed by the quantification of Lyve-1 labelled lymphatic vessels of the entire cornea (*P* = 0.3064) (Fig. 5). Structural features of the lymphatic network developed in the PAI-1 WT or PAI-1^−/−^ mice were also similar since all parameters analysed by computerized image analysis were identical [26]: the end-point density reflecting the number of vessels (*P* = 0.2715), the node density representing the number of branchings (*P* = 0.4526) and the length density corresponding the total length of the vessels (*P* = 0.3998).

## Discussion

The plasminogen activation system is largely implicated in pathological angiogenic processes. The genetic deficiency of PAI-1 in mice is associated to impaired blood vascularisation in several experimental models [9], [12], [18] such as cancer [10], [13] and choroidal angiogenesis models [11], [14]. The pivotal role of PAI-1 in pathological angiogenesis led to the expectation that this protease inhibitor regulates also lymphangiogenesis. Surprisingly, we previously reported that neither PAI-1 deficiency nor a pharmacological inhibitor of serine proteases directly affect the endothelial cell sprouting from thoracic duct explants in the lymphatic ring assay [29]. Nevertheless, this finding does not exclude a putative role of PAI-1 *in vivo* depending on the *in vivo* microenvironment and for instance, on an inflammatory reaction which is often associated with lymphangiogenesis. To address this important issue, we applied to PAI-1 deficient mice two models of breast cancer and two models of inflammation-related lymphangiogenesis. We found that PAI-1 is not essential for pathological lymphangiogenesis.

We first used a orthotopic graft model of VEGF-C overexpressing-mammary carcinoma cells (MCF7). VEGF-C has been indeed reported to promote tumor growth in SCID [22] and nude mice [23]. Tumor lymphangiogenesis was increased in VEGF-C MCF7 tumors injected in nude mice and lymph node metastasis were found more frequently [23]. Similar results were reported with VEGF-C overexpressing MDA-MB-435 [30]. In the present study, MCF7 cells overexpressing or not VEGF-C were inoculated into RAG-1^−/−^ immunodeficient mice crossed with PAI-1 WT or PAI-1 deficient mice. In accordance with previous reports [22], [23], we confirmed the increased growth rate of VEGF-C expressing tumors. The pro-tumoral effect of VEGF-C was previously attributed to a better oxygenation due to a slight angiogenic response or a decreased intratumoral pressure because of the increased number of lymphatic vessels. It is worth noting that the mice background and the immunodeficiency rate are essential factors influencing the lymph node dissemination. Indeed, the propensity of VEGF-C expressing cells to disseminate into lymph node was higher in nude mice [23], [30] than in SCID mice [22] or RAG-1^−/−^ mice (the present study). Since these mice differ in their B-lymphocyte status, it suggests that B lymphocytes might contribute to lymph node dissemination of cancerous cells. Accordingly, the requirement of B-lymphocytes was also observed in a lymphangiogenesis model of mycoplasma infection of the pulmonary tract [31].

In agreement with previous studies, PAI-1 deficiency was associated with decreased tumor development [10], [18]. We further analysed the lymphatic invasion of these tumors and their dissemination into lymph nodes. Although VEGF-C expression led to an enhancement of lymphatic vessel numbers, no difference was observed in PAI-1 WT and PAI-1^−/−^ mice. Moreover, both genotypes showed a similar rate of lymph node metastasis. These data clearly demonstrate that PAI-1 is not implicated in tumoral lymphangiogenesis. Moreover, our data are in line with a previous study on PyMT transgenic mice showing that the primary tumor growth was not significantly affected by PAI-1 deficiency and neither was the lung metastatic burden [21]. We now demonstrate that PAI-1 is dispensable for tumoral lymphangiogenesis by using the PyMT and PAI-1 double transgenic mice.

Knowing that inflammation influences cancer progression [32], [33] and that lymphangiogenesis and inflammation processes are closely related, we applied a model of lymphangioma to PAI-1^−/−^ mice. This system consists in a benign hyperplasia of lymphatic vessels induced by the injection of Freund adjuvant and is often used to isolate lymphatic endothelial cells [24], [34]. In this system, the inflammatory reaction induced by Freund adjuvant relies on the recruitment of leukocytes by cytokines secreted by cells of the peritoneum [35]. In PAI-1 deficient mice, we observed a macroscopic decrease of the lymphangioma formation as compared to PAI-1 WT mice. This effect could be ascribed to a reduction of fibrosis rather than to a decrease in lymphatic vessel recruitment. Accordingly, PAI-1 deficiency slowed down the fibrotic reaction in different models [36], [37] by accelerating plasmin-mediated proteolysis [36] or by influencing macrophage or myofibroblast recruitment [37]. The lack of PAI-1 effect on inflammation related lymphangiogenesis was further confirmed by similar injury-induced corneal lymphangiogenesis observed in PAI-1^−/−^ and PAI-1 WT mice. The increased lymphatic vessel size observed in lymphangioma of PAI-1^−/−^ mice is intriguing. However, note that this variation in vessel structure is associated with a reduction of matrix deposition which may influence vessel branching. Studies on mammary gland morphogenesis revealed that the collagen deposition inhibition reduced developing tubular structure bifurcations [38]. The matrix proteolytic breakdown could compromise the scaffold mechanical integrity necessary to counter endothelial cells-generated forces during the tube formation process [39], [40]. Thus, the difference in vessel structure likely relies on PAI-1-regulated fibrotic response rather than on a direct effect of PAI-1 on lymphangiogenesis.

The present study using genetic approaches provide for the first time evidences that in contrast to its pivotal role in pathological angiogenesis, PAI-1 is dispensable in pathological lymphangiogenesis in tumoral situations as well as in inflammatory disorders. This clearly demonstrates that distinct molecular pathways govern angiogenesis and lymphangiogenesis and that PAI-1 plays distinct roles in the remodelling of both circulation systems in pathological conditions. Providing that PAI-1 antagonists are used to inhibit angiogenesis [41], our results reveal that this approach will not have any effect on lymphangiogenesis. Although PAI-1 is dispensable for lymphangiogenesis, it is worth noting that other proteolytic systems are mandatory for this process and particularly matrix metalloproteases such as the MMP-2 whose deficiency impairs lymphangiogenesis in *in vitro* and *in vivo* models [29].
